# Zoledronic acid exacerbates inflammation through M1 macrophage polarization

**DOI:** 10.1186/s41232-018-0074-9

**Published:** 2018-06-23

**Authors:** Junya Kaneko, Toshinori Okinaga, Hisako Hikiji, Wataru Ariyoshi, Daigo Yoshiga, Manabu Habu, Kazuhiro Tominaga, Tatsuji Nishihara

**Affiliations:** 1 Division of Infections and Molecular Biology, Department of Health Promotion, Kyushu Dental University, Kitakyushu, Fukuoka, 803-8580 Japan; 2 School of Oral Health Sciences, Kyushu Dental University, Kitakyushu, Fukuoka, 803-8580 Japan; 3 Division of Oral and Maxillofacial Surgery, Department of Science of Physical Functions, Kyushu Dental University, Kitakyushu, Fukuoka, 803-8580 Japan

**Keywords:** Zoledronic acid, Macrophage polarization, Inflammation

## Abstract

**Background:**

Zoledronic acid (Zol), one of the bisphosphonates, is frequently utilized for the treatment of osteoporosis and bone metastasis. However, the onset of medication-related osteonecrosis of the jaw (MRONJ) following dental treatments has become a serious issue. We reported previously that osteonecrosis can be induced by Zol and lipopolysaccharide (LPS) in vivo, suggesting the involvement of Zol in inflammation. Macrophages are divided into M1/M2 macrophages. M1 macrophages are involved in the induction and exacerbation of inflammation and express proinflammatory mediators including interleukin (IL)-1. On the other hand, M2 macrophages are associated with anti-inflammatory reactions through the expression of anti-inflammatory cytokines, such as IL-10. In the present study, we clarified the effects of Zol on M1/M2 macrophage polarization in vitro.

**Methods:**

Human monocytic THP-1 cells were polarized to macrophage-like cells by phorbol 12-myristate 13-acetate (PMA), and, after culturing for an additional 24 h with or without Zol, then polarized to M1 macrophages by LPS or to M2 macrophages by IL-4. Cell viability was examined by the WST-8 assay. Gene expression was confirmed by the real-time polymerase chain reaction. Protein expression was detected by western blotting and enzyme-linked immunosorbent assays.

**Results:**

Zol treatment upregulated the expression of IL-1β mRNA and protein through NLRP3 inflammasome activation in LPS-treated THP-1 cells. Zol treatment did not affect the expression of IL-10, IL-1ra, or CD206 in IL-4-treated THP-1 cells.

**Conclusions:**

Zol enhanced LPS-induced M1, but not M2, macrophage polarization through the NLRP3 inflammasome-dependent pathway, resulting in the production of inflammatory cytokines in THP-1 cells.

## Background

Nitrogen-containing bisphosphonates, including zoledronic acid (Zol), are widely used as anti-bone-resorptive agents, primarily for the treatment of osteoporosis, Paget’s disease of the bone, multiple myeloma, hypercalcemia due to malignancy, and other bone-resorptive diseases. The onset of medication-related osteonecrosis of the jaw (MRONJ) has become a serious issue. Dental treatment such as tooth extraction triggers the MRONJ in the patients taking anti-bone-resorptive agents. The clinical symptoms often seen in MRONJ including pain, swelling, paresthesia, suppuration, and intraoral/extraoral fistula continue for a long time [1]. Marx et al. reported the first case of osteonecrosis of the jaw (ONJ) in 2003 [2, 3]. Since then, the number of patients with ONJ has been increasing yearly. Bisphosphonates are one of the most well-known agents that cause ONJ [4]. We reported that the combined use of Zol and lipopolysaccharide (LPS) in vivo induced ONJ and osteonecrosis of the femur in rats, suggesting that Zol is involved in the inflammatory response during the progression of MRONJ [5–7].

Macrophages are derived from monocytes and move out into extravascular tissues under inflammatory or non-inflammatory conditions, playing different roles according to their surrounding environment [8]. Oral macrophages also play important roles in the inflammatory response, as well as in signaling to resolve inflammation, and promote healing and regeneration [9].

Macrophages are divided into M1 and M2 macrophage types [10]. While investigating the factors that regulate macrophage arginine metabolism, Mills et al. found that macrophages activated in mouse strains with T helper type (Th)1 and Th2 backgrounds differed qualitatively in their ability to respond to the classic stimulation of interferon (IFN)-γ or LPS or both and defined an important metabolic difference in the pathway. They proposed that these be termed M1 and M2 macrophage responses [11]. Macrophages are polarized into the M1 macrophages, when exposed to classical activators such as LPS and IFN-γ [12]. Macrophages are polarized into the M2 from when exposed to alternative activators such as interleukin (IL)-4 or IL-13 [12]. M1-polarized macrophages produce pro-inflammatory cytokines, such as IL-1β, and infiltrate into injured tissues soon after damage [13]. M2-polarized macrophages are major resident macrophages and appear at late stages of repair and remodeling in injured tissue [14]. In our previous study, we have revealed that Zol activated NF-κB by enhancing IκB-α degradation suggesting the involvement of M1-polarized macrophages [15]. Therefore, we hypothesized that Zol might be involved in M1 but not M2 macrophage polarization, resulting in the inflammatory function in MRONJ. It is interesting to know the role of Zol in M1- or M2-polarized macrophages to probe into the cause of Zol-induced MRONJ. Therefore, in the present study, we investigated the effect of Zol on M1/M2 macrophage polarization in vitro and revealed that Zol and LPS synergistically enhanced proinflammatory character of THP-1 cells via activation of inflammasome.

## Methods

### Cell culture conditions

The human monocytic cell line, THP-1 (JCRB0112.1; JCRB Cell Bank, Osaka, Japan), was cultured in RPMI 1640 medium (Gibco Laboratories, Grand Island, NY, USA), supplemented with 5% heat-inactivated fetal bovine serum (FBS; CORNING, NY, USA), penicillin G (100 U/ml) (Nacalai Tesque, Kyoto, Japan), and streptomycin (100 mg/ml; Wako Pure Chemical Industries, Osaka, Japan) at 37 °C with 5% CO_2_. THP-1 cells were seeded at 2 × 10^6^ cells/well in six-well plates (Iwaki, Chiba, Japan) and cultured in RPMI 1640 medium containing 5% FBS and 100 ng/ml phorbol 12-myristate 13-acetate (PMA) (Sigma-Aldrich, St. Louis, MO, USA). After culturing overnight, cells were washed with phosphate-buffered saline (PBS; pH 7.2). THP-1 cells were then treated with or without Zol (10 μM; Sigma-Aldrich). After culturing for an additional 24 h with or without Zol, LPS from *Escherichia coli* (100 ng/ml; Sigma–Aldrich) or IL-4 (50 ng/ml; R&D Systems, Minneapolis, MN, USA) were added.

### Reagents

Anti-IL-1β, anti-apoptosis-associated speck-like protein containing a caspase recruitment domain (ASC), and anti-cluster of differentiation (CD) 206 polyclonal antibodies were obtained from Santa Cruz Biotechnology (Santa Cruz, CA, USA). Anti-caspase-1 p-20 and NOD-like receptor protein 3 (NLRP3) monoclonal antibodies were obtained from Adipogen Life Sciences (San Diego, CA, USA). An anti-β-actin monoclonal antibody was obtained from Sigma–Aldrich. In some experiments, 5 mM ATP (Sigma–Aldrich) was applied to LPS-treated THP-1 cells for 30 min before collecting samples.

### WST-8 assay

Cell viability was determined using the tetrazolium salt, WST-8 (2-(2-methoxy-4-nitrophenyl)-3-(4-nitrophenyl)-5-(2,4-disulfophenyl)-2H-tetrazolium, monosodium salt) (Dojindo Laboratories, Kumamoto, Japan). THP-1 cells (4 × 10^5^/well) were seeded in 96-well plates in RPMI 1640 containing 5% FBS and 100 ng/ml PMA. After culturing overnight, cells were washed with PBS (pH 7.2) twice and then exposed to Zol for 48 h. WST-8 solution (10 μl) was then added to each well, followed by incubation for 2 h. Absorbance at 450 nm was measured using a Multiscan JX microplate reader (Thermo Electron, Kanagawa, Japan).

RNA extraction and real-time reverse transcriptase-polymerase chain reaction (RT-PCR) analysis.

THP-1 cells were harvested, centrifuged at 4 °C, and stored at – 80 °C. RNA was extracted from cell pellets using a Cica Geneus RNA Prep Kit (KANTO CHEMICAL, Tokyo, Japan) according to the manufacturer’s instructions. Total RNA was used for cDNA synthesis using ReverTra Ace qPCR RT Master Mix (TOYOBO, Osaka, Japan) according to the manufacturer’s instructions. Primers for real-time RT-PCR were designed using Primer Express 3.0 software (Applied Biosystems, Foster City, CA, USA). Reactions were prepared using Brilliant III Ultra-Fast SYBR Green QPCR Master Mix With Low ROX (Agilent Technologies, Santa Clara, CA, USA). Detection was performed with an AriaMx Real-Time PCR System (Agilent Technologies). Relative changes in gene expression were calculated by the comparative CT (ΔΔCT) method. Total cDNA abundance between samples was normalized using primers specific for the β-actin gene.

The primers used for real-time RT-PCR were as follows: human *IL-1β* (GenBank accession no. NM_000576), forward 5′-TCAGCCAATCTTCATTGCTCAA-3′ and reverse 5′-TGGCGAGCTCAGGTACTTCTG-3′; human *IL-1ra* (GenBank accession no. NM_173842), forward 5′-CTCCTCTTCCTGTTCCATTCAG-3′ and reverse 5′-AAGGTCTTCTGGTTAACATCCC-3′; human *IL-10* (GenBank accession no. NM_000572), forward 5′-GCTGGAGGACTTTAAGGGTTAC-3′ and reverse 5′-GATGTCTGGGTCTTGGTTCTC-3′; human *CD206* (GenBank accession no. NM_002438), forward 5′-GGACGTGGCTGTGGATAAAT-3′ and reverse 5′-ACCCAGAAGACGCATGTAAAG-3′; and human *β-actin* (GenBank accession no. E0 1094), forward 5′-GCGCGGCTACAGCTTCA-3′ and reverse 5′-CTTAATGTCACGCACGATTTCC-3′.

### Western blotting analysis

Following treatments, cells were lysed in sodium dodecyl sulfate (SDS) lysis buffer (50 mM Tris–HCl and 2% SDS; pH 6.8) containing a protease inhibitor mixture (Nacalai Tesque). Then, the protein content of the samples was determined using a protein assay reagent (Bio-Rad, Hercules, CA, USA). Protein samples (20 μg) were subjected to electrophoresis on SDS-polyacrylamide gels and electroblotted onto polyvinylidene fluoride membranes. The membranes were blocked for 30 min with Blocking One (Nacalai Tesque) and incubated with the primary antibodies for 2 h. After washing with Tris-buffered saline containing 0.1% Tween 20 (TBS-T), the membranes were incubated with the secondary antibody for 1 h. After washing with TBS-T, immunodetection was performed using the ECL Prime Western Blotting Detection Reagent (GE Healthcare, Little Chalfont, UK) and a ChemiDoc XRS Plus imaging system (Bio-Rad). Densitometric analysis of protein bands in the western blots was performed by Image Lab software (Bio-Rad). Data were normalized to β-actin expression and are expressed as means ± standard deviation (SD) of triplicate cultures.

### Enzyme-linked immunosorbent assay (ELISA) analysis

Supernatants from THP-1 cells were collected at 0–48 h following LPS treatments. Secreted cytokine levels were assessed using human IL-1β/IL-1F2 Quantikine HS and human IL-1ra/IL-1F3 Quantikine ELISA kits (R&D Systems) according to the manufacturer’s instructions.

### Silencing of ASC expression by specific siRNA

siRNA targeting was used to knock down ASC expression in THP-1 cells. siRNAs against human ASC and siRNA control were purchased from Nacalai Tesque. A NEPA21 Super Elec-troporator (Nepa Gene Co., Ltd., Chiba, Japan) was used to deliver siRNA into cells according to the manufacturer’s instructions. In brief, 1 × 10^6^ cells were suspended in 100 μL of RPMI 1640 and transfected with siRNA at a final concentration of 300 nM.

### Statistical analysis

All data are expressed as means ± SD of three individual experiments with similar results obtained in each experiment. Statistical differences were determined using unpaired Student’s *t* test. A value of *P* < 0.05 was considered as statistically significant.

## Results

### Zol enhanced IL-1β expression and the secretion of mature IL-1β during M1 macrophage differentiation

**Fig. 1:**
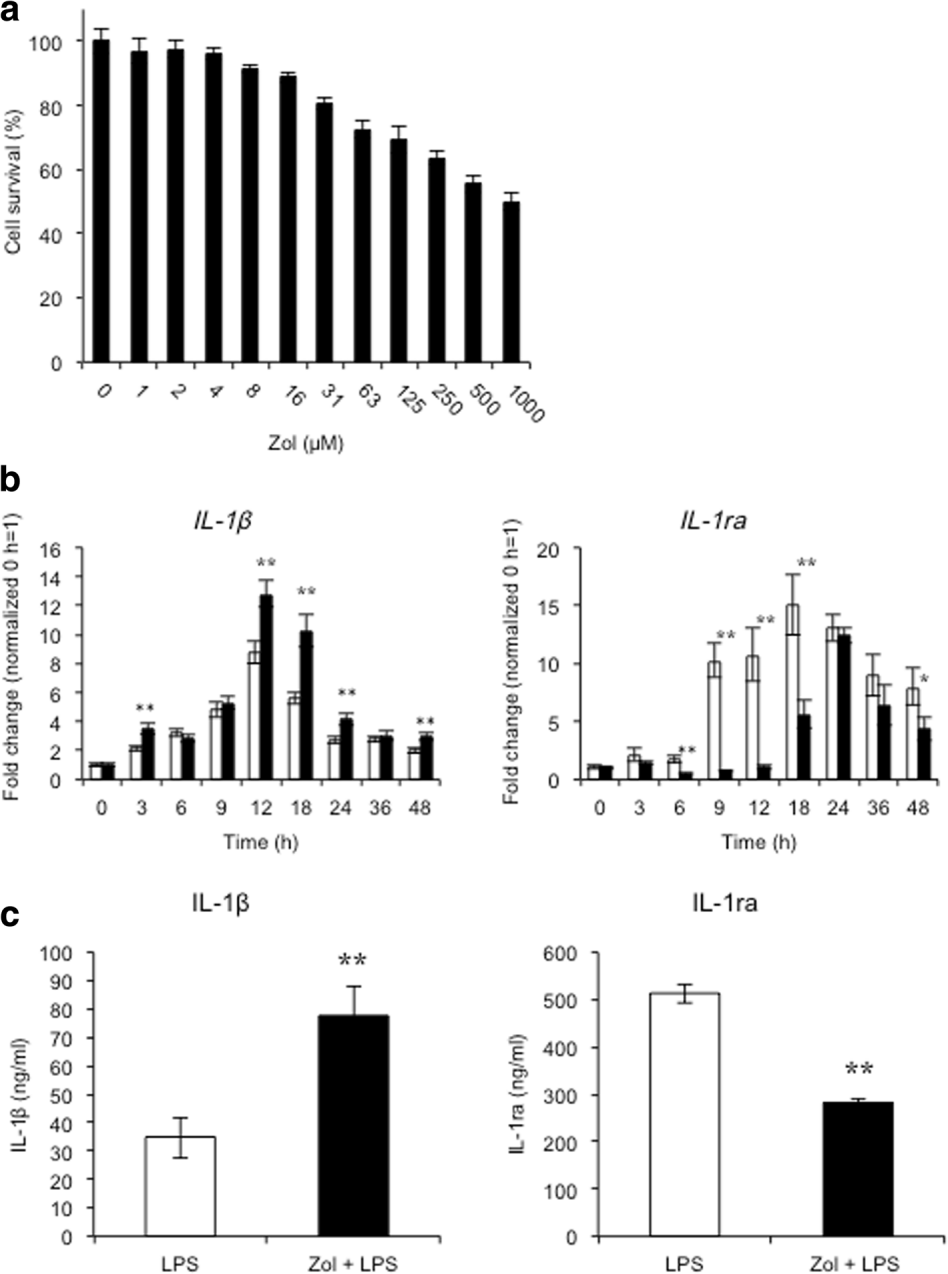
Effects of zoledronic acid on the expression of M1/M2 macrophage markers in LPS-treated THP-1 cells. THP-1 cells were treated overnight with 100 ng/ml PMA, then washed and incubated for 24 h. **a** Cell viability was assessed using a WST-8 assay after treatment with Zol (1–1000 nM) for 48 h. **b** THP-1 cells were incubated with or without Zol for 24 h and then treated with 100 ng/ml LPS. Open bars represent LPS-treated THP-1 cells; filled bars represent Zol and LPS-treated THP-1 cells. Zol upregulated the expression of IL-1β mRNA and downregulated the expression of IL-1ra mRNA in LPS-treated THP-1 cells (* P < 0.05, ** P < 0.01 vs. cells treated with LPS alone). **c** ATP was applied to LPS-treated cells for 30 min before collecting samples. The secretion of IL-1β at 18 h and IL-1ra at 36 h was detected by ELISA. Zol upregulated the secretion of IL-1β and downregulated the secretion of IL-1ra in LPS-treated THP-1 cells (** P < 0.01 vs. cells treated with LPS alone)

THP-1 cells were exposed to Zol for 48 h after PMA treatment. From the result of the WST-8 assay (Fig. 1a), 10 μM Zol was used for subsequent experiments to minimize its toxic effects on cell viability [15, 16].

LPS is known to polarize monocytes/macrophages to M1 macrophages [17]. Zol upregulated the expression of IL-1β mRNA, one of the major inflammatory cytokines, as well as M1 macrophage markers, in LPS-treated THP-1 cells. In contrast, Zol downregulated the expression of interleukin-1 receptor antagonist (IL-1ra) mRNA, which is a naturally occurring cytokine preventing the biologic response to IL-1 [18], in LPS-treated THP-1 cells (Fig. 1b; *P < 0.05, **P < 0.01).

Zol upregulated the secretion of mature IL-1β, an active form of IL-1β that is cleaved from an inactive precursor, in LPS-treated THP-1 cells. On the other hand, Zol downregulated the secretion of IL-1ra in LPS-treated THP-1 cells (Fig. 1c; **P < 0.01).

### Zol enhanced the expression of inflammasome-associated molecules during M1 macrophage differentiation

**Fig. 2:**
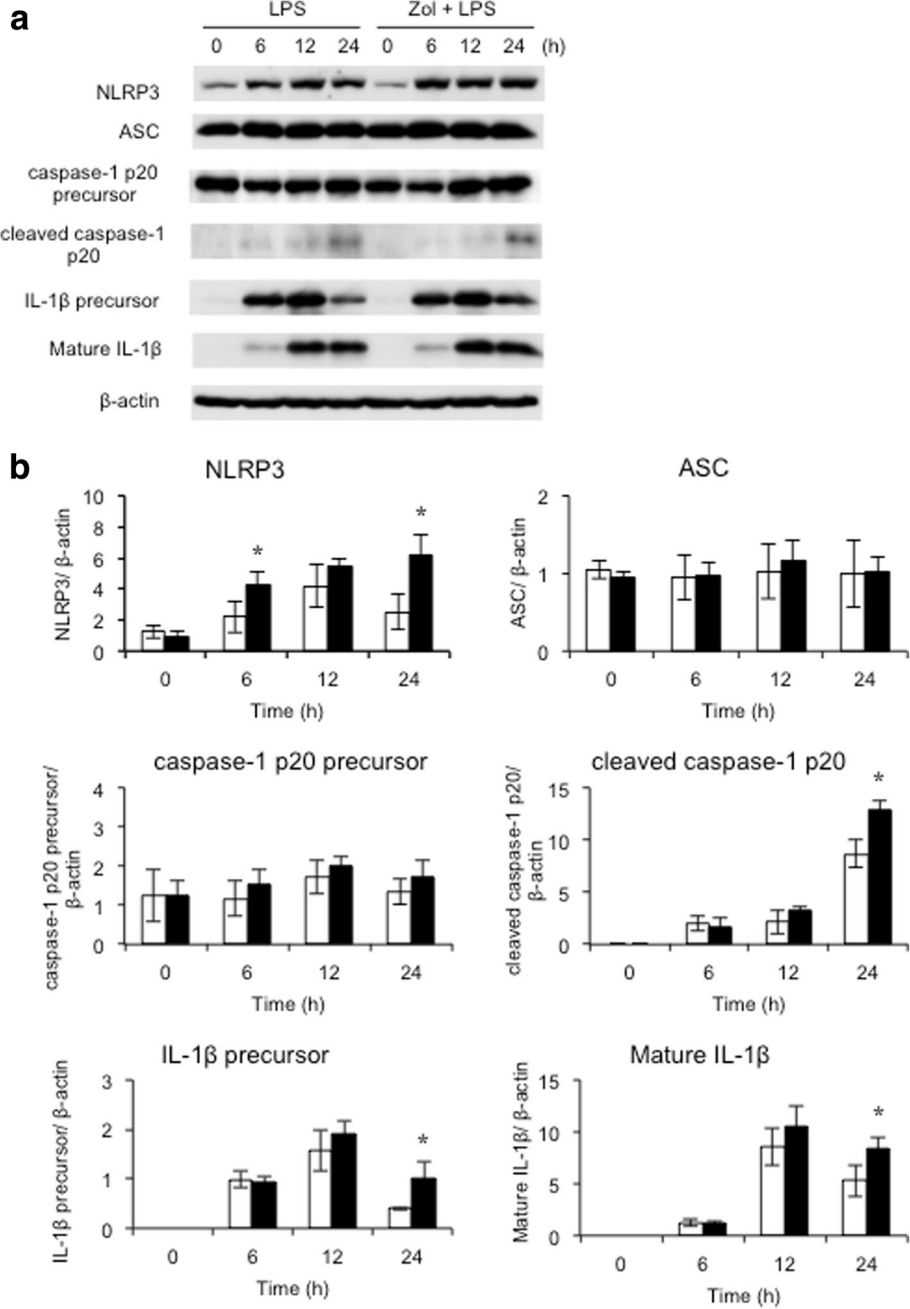
Effects of zoledronic acid on the expression of inflammasome-associated proteins. THP-1 cells were treated overnight with 100 ng/ml PMA, washed with PBS, and incubated with or without Zol for 24 h. THP-1 cells were then treated with 100 ng/ml LPS. ATP was applied to LPS-treated cells for 30 min before collecting samples. **a** Protein expression was detected by western blotting. **b** Band intensities were measured by scanning densitometry. Data were normalized to β-actin expression. Open bars represent LPS-treated THP-1 cells; filled bars represent Zol and LPS-treated THP-1 cells. Zol promoted the expression of NLRP3, cleaved caspase-1 p20, IL-1β precursor, and mature IL-1β proteins in LPS-treated THP-1 cells (*P < 0.05 vs. cells treated with LPS alone)

The NLRP3 inflammasome is crucial for the formation of mature IL-1β. Therefore, we investigated the expression of NLRP3 inflammasome-associated proteins in LPS-treated THP-1 cells by western blot analysis. Among NLRP3 inflammasome-associated molecules, several proteins, including NLRP3, caspase-1 p20 precursor, cleaved caspase-1 p20, IL-1β precursor, and mature IL-1β, and ASC, were investigated. Zol upregulated the expression of NLRP3, cleaved caspase-1 p20, IL-1β precursor, and mature IL-1β in LPS-treated THP-1 cells. Caspase-1 p20 precursor and ASC were constitutively expressed throughout the experimental time (Fig. 2).

**Fig. 3:**
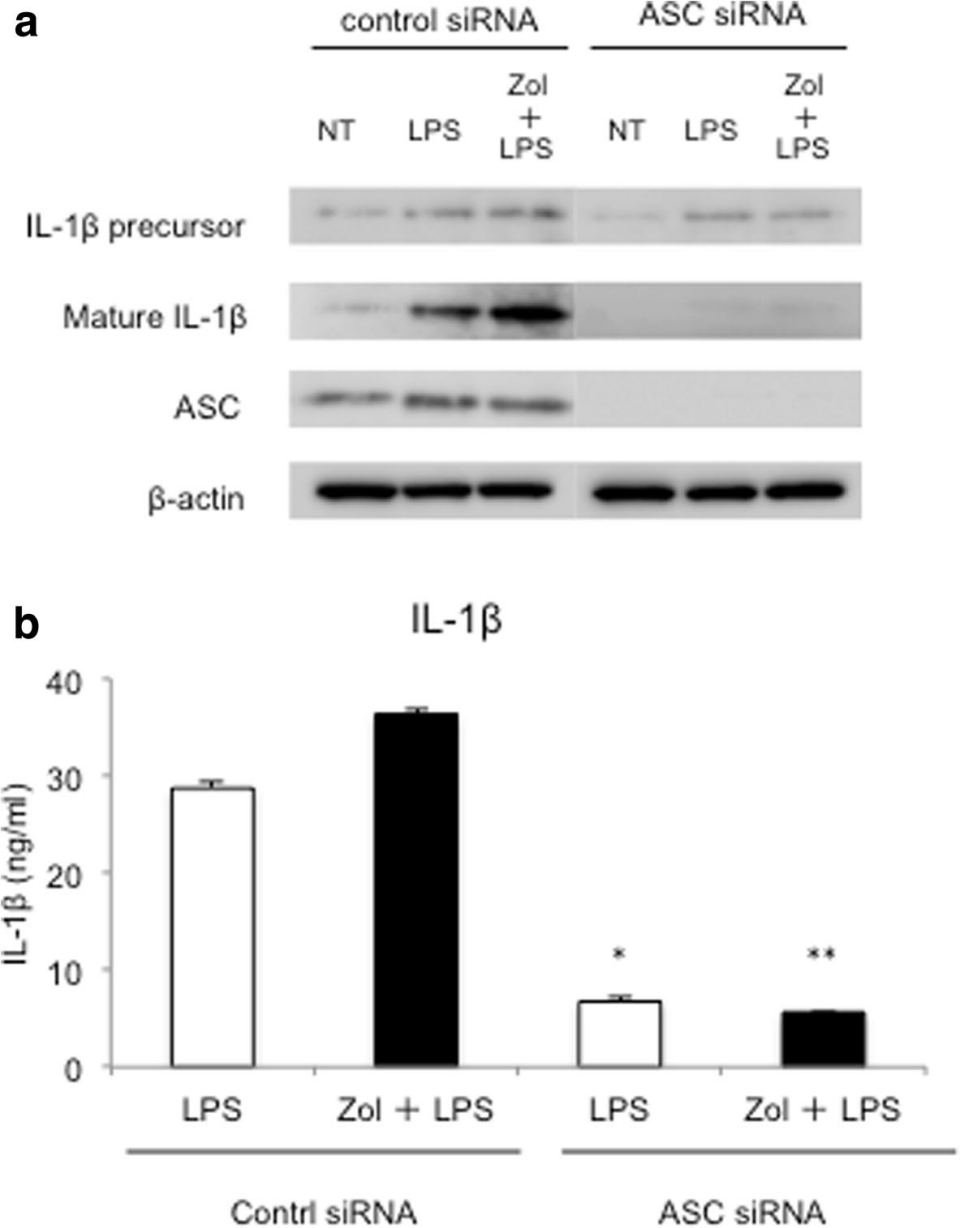
Role of NLRP3 in IL-1β secretion induced in LPS-treated THP-1 cells. THP-1 cells were treated with siRNA against ASC, incubated overnight with 100 ng/ml PMA, washed with PBS, and incubated further with or without Zol for another 24 h. THP-1 cells were then treated with 100 ng/ml LPS. ATP was applied to LPS-treated cells for 30 min before collecting samples. **a** Protein expression was detected by western blotting. Silencing of ASC downregulated the expression of the mature IL-1β in LPS-treated THP-1 cells. **b** The secretion of IL-1β was detected by ELISA. Open bars represent LPS-treated THP-1 cells; filled bars represent Zol and LPS-treated THP-1 cells. Silencing of ASC reduced the secretion of IL-1β in LPS-treated THP-1 cells (*P < 0.01 vs. control siRNA THP-1 cells treated with LPS alone, ** P < 0.01 vs. control siRNA THP-1 cells treated with Zol and LPS)

To show the role of NLRP3 inflammasome in IL-1β expression and secretion of IL-1β in LPS-treated THP-1 cells, loss of function study of a NLRP3 inflammasome-associated molecule, ASC, was performed by siRNA. Silencing of ASC downregulated the protein expression of the mature IL-1β (Fig. 3a). Furthermore, silencing of ASC reduced the amount of secreted IL-1β (Fig. 3b).

### Zol had no effect on mRNA and protein expression of M2 macrophage-related molecules during M2 macrophage differentiation

**Fig. 4:**
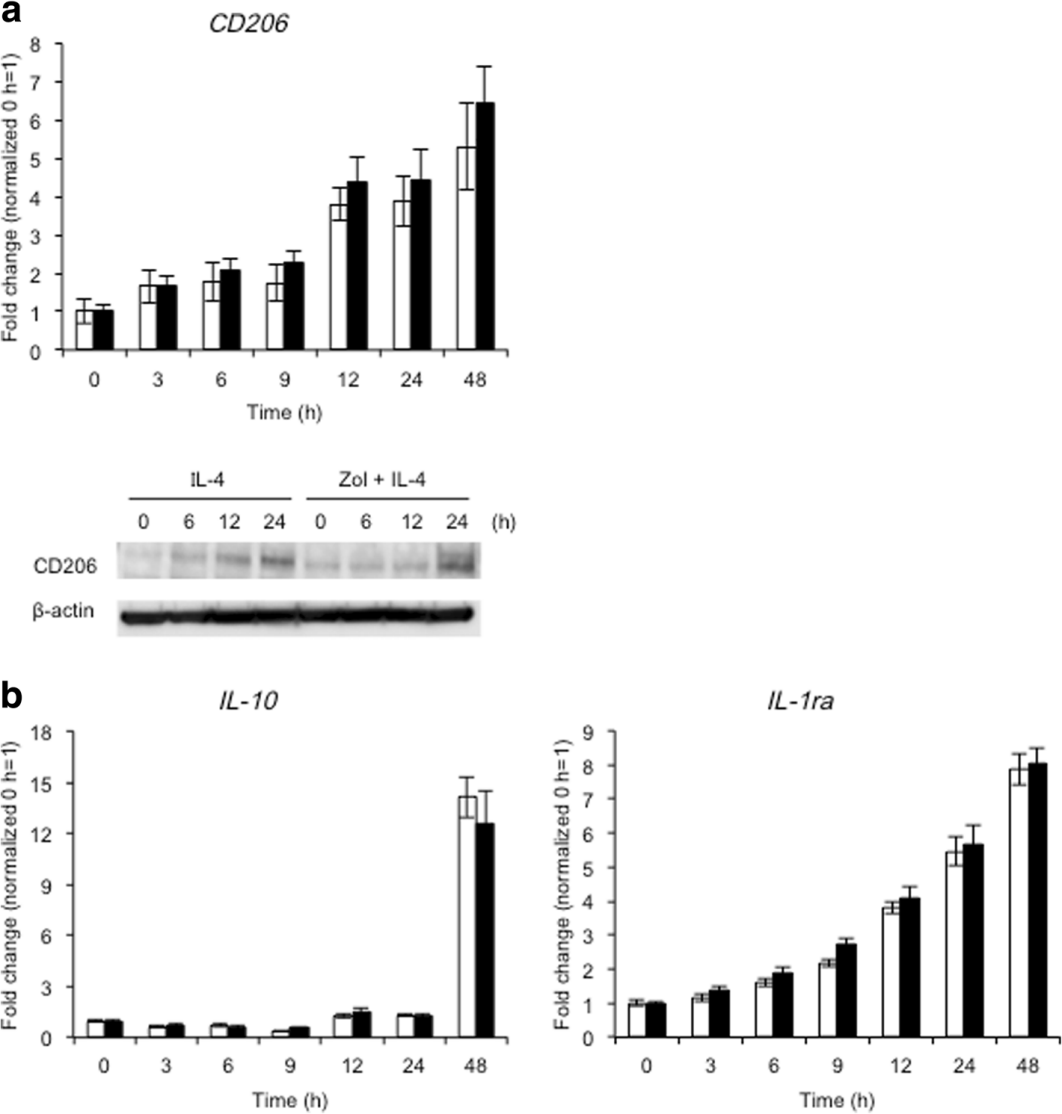
Effects of zoledronic acid on mRNA and protein expression of M2 macrophage markers in IL-4-treated THP-1 cells. THP-1 cells were treated overnight with 100 ng/ml PMA, washed with PBS, and incubated with or without Zol for 24 h. THP-1 cells were then treated with 50 ng/ml IL-4. mRNA expression was detected by the real-time RT-PCR. Protein expression was detected by western blotting. Open bars represent IL-4-treated THP-1 cells; filled bars represent Zol and IL-4-treated THP-1 cells. **a** Zol did not affect the mRNA and protein expression of CD206 in IL-4-treated THP-1 cells. **b** Zol did not affect the mRNA expression of IL-10 and IL-1ra in IL-4-treated THP-1 cells

IL-4 polarizes monocytes/macrophages to M2 macrophages [19]. Zol did not have any effect on the mRNA and protein expression of CD206, a well-known M2 macrophage marker [20], in IL-4-treated THP-1 cells (Fig. 4a). Furthermore, Zol had no effect on the expression of IL-10 mRNA, one of the cytokines produced by M2 macrophages, and IL-1ra mRNA, a highly expressed molecule in M2 macrophages, in IL-4-treated THP-1 cells (Fig. 4b) [12].

## Discussion

Zol enhances the production of proinflammatory cytokines [15, 21, 22] suggesting that Zol polarizes macrophages toward an M1 phenotype. In the current study, we showed that Zol upregulated the expression of IL-1β mRNA and protein, and downregulated the expression of IL-1ra mRNA and protein, during M1 macrophage differentiation (Fig. 1). We have shown that Zol polarizes macrophages toward an M1 phenotype, but not an M2 phenotype and that Zol and LPS synergistically enhance proinflammatory character of THP-1 cells via activation of inflammasome.

In in vitro experiments, concentration of Zol at more than 10 μM is commonly used to investigate its effect on cells [15, 16, 23]. Clinically, the serum concentration of Zol is about 1.5 μM [24]. However, the serum concentration of Zol does not necessarily reflect the concentration of this compound affecting the cells in vivo. Therefore, 10 μM Zol was used for our experiments.

LPS stimulates inflammation through the production of cytokines such as IL-1β, finally resulting in the production of anti-inflammatory molecules like IL-1ra [25]. Therefore, it is not surprising that LPS upregulates the expression of IL-1ra during M1 macrophage differentiation (Fig. 1). On the other hand, IL-1ra also serves as a marker of the anti-inflammatory response [26]. In the current study, Zol suppressed the expression of IL-1ra in LPS-treated THP-1 cells as compared with Zol-non-treated and LPS-treated cells. These results suggest that Zol extinguished the anti-inflammatory response in THP-1 cells. Importantly, Zol had no effect on mRNA and protein expression of M2 macrophage-related molecules such as IL-10, IL-1ra, and CD206 during M2 macrophage differentiation induced by IL-4 treatment (Fig. 4). These results clearly show that Zol prompts the differentiation of M1 but not M2 macrophages.

Various signaling pathways are reportedly involved in Zol-induced macrophage polarization [15, 21, 22]. Among these, the inflammasome is a large intracellular protein complex that recruits and activates caspase-1 which, in turn, cleaves the proform of IL-1β to its biologically active and secreted form [27]. The NLRP3 inflammasome is critical for the formation of mature IL-1β [28]. NLRP3 is the intracellular receptor of inflammasomes, and ATP activates NLRP3 [29, 30]. It is also well-known that ATP stimulates the secretion of mature IL-1β [31]. Because Zol treatment upregulated the mRNA and protein expression of IL-1β in our study, we investigated the effects of Zol on NLRP3 inflammasome activation by using ATP as a second signal to NLRP3. Zol upregulated the protein expression of NLRP3 and caspase-1 in LPS-treated THP-1 cells (Fig. 2). Silencing of ASC is reported to inhibit IL-1β release in LPS-treated THP-1 cells [32]. We have found that silencing of ASC downregulated the protein expression of the mature IL-1β and reduced the secretion of IL-1β in LPS-treated THP-1 cells (Fig. 3). These results suggest that NLRP3 inflammasome molecules are involved in the maturation of IL-1β and the secretion of IL-1β. In total, our results demonstrate that Zol upregulates M1 macrophage differentiation through the NLRP3 inflammasome-dependent pathway. Additional research is required to investigate how inflammasome receptors other than NLRP3 may play a role in Zol-induced M1 macrophage polarization.

## Conclusions

We have shown directly that Zol enhanced LPS-induced M1, but not M2, macrophage polarization through the NLRP3 inflammasome-dependent pathway, resulting in the production of inflammatory cytokines in THP-1 cells.

## Abbreviations

ASC: Apoptosis-associated speck-like protein containing a caspase recruitment domain
ATP: Adenosine triphosphate
CD206: Cluster of differentiation 206
ELISA: Enzyme-linked immunosorbent assay
FBS: Fetal bovine serum
IL-10: Interleukin-10
IL-13: Interleukin-13
IL-1ra: Interleukin-1 receptor antagonist
IL-1β: Interleukin-1β
IL-4: Interleukin-4
LPS: Lipopolysaccharide
mRNA: Messenger ribonucleic acid
NLRP3: Nod-like receptor 3
PBS: Phosphate-buffered saline
PMA: Phorbol 12-myristate 13-acetate
RT-PCR: Reverse transcription-polymerase chain reaction
Zol: Zoledronic acid
